# Glycolytic enzyme PFKFB3 regulates sphingosine 1-phosphate receptor 1 in proangiogenic glomerular endothelial cells under diabetic condition

**DOI:** 10.1152/ajpcell.00261.2023

**Published:** 2023-10-02

**Authors:** Baixue Yu, Kaiyuan Shen, Tingting Li, Jiawei Li, Mei Meng, Wenjie Liu, Qunye Tang, Tongyu Zhu, Xin Wang, Susan W. S. Leung, Yi Shi

**Affiliations:** ^1^Institute of Clinical Science, Zhongshan Hospital, Fudan University, Shanghai, People’s Republic of China; ^2^Key Laboratory of Organ Transplantation, Zhongshan Hospital, Fudan University, Shanghai, People’s Republic of China; ^3^Department of Neurology, Zhongshan Hospital, Fudan University, Shanghai, People’s Republic of China; ^4^Department of Urology, Zhongshan Hospital, Fudan University, Shanghai, People’s Republic of China; ^5^Department of Pharmacology and Pharmacy, Li Ka Shing Faculty of Medicine, The University of Hong Kong, Hong Kong SAR, People’s Republic of China

**Keywords:** diabetic nephropathy, glomerular endothelial cells, microRNA-590-3p, phosphofructokinase/fructose bisphosphatase 3, sphingosine 1-phosphate receptor 1

## Abstract

Glomerular angiogenesis is a characteristic feature of diabetic nephropathy (DN). Enhanced glycolysis plays a crucial role in angiogenesis. The present study was designed to investigate the role of glycolysis in glomerular endothelial cells (GECs) in a mouse model of DN. Mouse renal cortex and isolated glomerular cells were collected for single-cell and RNA sequencing. Cultured GECs were exposed to high glucose in the presence (proangiogenic) and absence of a vascular sprouting regimen. MicroRNA-590-3p was delivered by lipofectamine in vivo and in vitro. In the present study, a subgroup of GECs with proangiogenic features was identified in diabetic kidneys by using sequencing analyses. In cultured proangiogenic GECs, high glucose increased glycolysis and phosphofructokinase/fructose bisphosphatase 3 (PFKFB3) protein expression, which were inhibited by overexpressing miRNA-590-3p. Mimics of miRNA-590-3p also increased receptor for sphingosine 1-phosphate (S1pR1) expression, an angiogenesis regulator, in proangiogenic GECs challenged with high glucose. Inhibition of PFKFB3 by pharmacological and genetic approaches upregulated S1pR1 protein in vitro. Mimics of miRNA-590-3p significantly reduced migration and angiogenic potential in proangiogenic GECs challenged with high glucose. Ten-week-old type 2 diabetic mice had elevated urinary albumin levels, reduced renal cortex miRNA-590-3p expression, and disarrangement of glomerular endothelial cell fenestration. Overexpressing miRNA-590-3p via perirenal adipose tissue injection restored endothelial cell fenestration and reduced urinary albumin levels in diabetic mice. Therefore, the present study identifies a subgroup of GECs with proangiogenic features in mice with DN. Local administration of miRNA-590-3p mimics reduces glycolytic rate and upregulates S1pR1 protein expression in proangiogenic GECs. The protective effects of miRNA-590-3p provide therapeutic potential in DN treatment.

**NEW & NOTEWORTHY** Proangiogenetic glomerular endothelial cells (GECs) are activated in diabetic nephropathy. High glucose upregulates glycolytic enzyme phosphofructokinase/fructose bisphosphatase 3 (PFKFB3) in proangiogenetic cells. PFKFB3 protects the glomerular filtration barrier by targeting endothelial S1pR1. MiRNA-590-3p restores endothelial cell function and mitigates diabetic nephropathy.

## Graphical abstract

**Figure:**
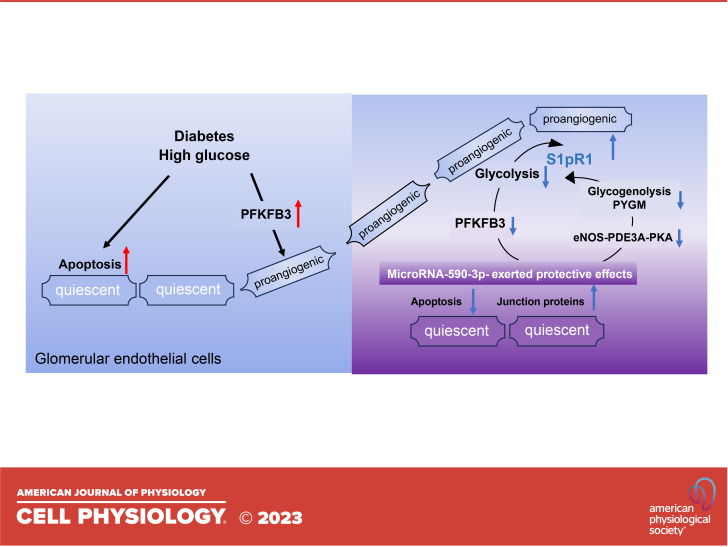

## INTRODUCTION

Diabetic nephropathy is a leading cause of chronic renal failure, shown as microalbuminuria in the early stage and impaired renal function as the disease progresses (1). Microalbuminuria in patients with diabetes implies a disruption of the glomerular filtration barrier composed of podocytes, glomerular endothelial cells (GECs), and the glomerular basement membrane. In addition to the critical involvement of podocytes (2), reduced fractions of fenestrated endothelial cells are observed in patients with diabetes with normoalbuminuria (3). It indicates that glomerular endothelial cells also play a role in initiating and developing diabetic nephropathy.

Using single-cell sequencing technology in human early diabetic nephropathy (GSE131882) (4) and glomerular cells of mice with diabetic nephropathy (GSE127235) (5), angiogenesis is a critical biological process (4–6). In addition to the well-studied roles of endothelial cells in macrocirculation, endothelial cells in microcirculation are the key to endothelial barriers by preventing vascular leakage. In the process of angiogenesis, endothelial cells in microcirculation are activated into the tip cell, shown as long and dynamic filopodia, and the elongated and proliferative stalk cell. Sprouting endothelial cells, both tip and stalk cells, have high mobility and low adherent capacity, resulting in vessel leakage until maturation.

Glycolysis is the energy-producing setting for vascular endothelial cells. In the process, hexokinase 2 (HK2), phosphofructokinase (PFK), and pyruvate kinase enzyme M2 (PKM2) are the rate-limiting enzymes (7, 8). Phosphofructokinase/fructose bisphosphatase 3 (PFKFB3), an allosteric activator of PFK, participates in regulating angiogenesis as well (9). It is reported that upregulation of PFKFB3 overbeats the Notch signals (10), whereas inhibiting PFKFB3 affects vascular endothelial growth factor receptor 2 (VEGFR2) activation in tip cells and normalizes disorganized angiogenesis (10). Abnormal glycolysis in endothelial cells has been reported in diabetes, atherosclerosis, pulmonary arterial hypertension, and arthritic joints (8). In addition to glycolysis, intracellular glucose levels are also regulated by processes of glycogenesis and glycogenolysis. Glycogen phosphorylase breaks glycogen, the storage form of intracellular glucose, and provides glucose for immediate requirements in glycogenolysis. It is reported that both endothelial nitric oxide synthase and glycogen phosphorylase expressions are reduced in diabetic myocardium (11), and arginine treatment restores endothelial cell integrity (11).

Sphingosine 1-phosphate receptor 1 (S1pR1) is highly expressed in endothelial cells and plays a critical role in regulating endothelial cell differentiation, migration, adhesion, angiogenesis, and vascular maturation. Tumor vessels lacking S1pR1 exhibit excessive vascular sprouting and blunted barrier function, whereas overexpressing S1pR1 reduces vessel branching and tortuosity (12). S1pR1 agonist normalizes the blood-brain barrier permeability to small molecules (13) and protects against ischemia-reperfusion injury by preventing tight junction protein redistribution in a rat stroke model (14). Therefore, it strongly indicates that S1pR1 is crucial in angiogenesis and endothelial permeability.

MicroRNAs (miRNA) regulate target genes by binding to 3′-untranslated regions of mRNA sequences. The presence of miRNA-590-3p is first reported in mouse hearts (15). MicroRNA-590-3p expression is reduced in endothelial cells stimulated with oxidized LDL (16), angiotensin II (17), and hypoxia (18). Overexpressing miRNA-590-3p downregulates inflammatory responses (19) and modulates angiogenesis (18), leading to the deceleration of diabetic retinopathy (19) and the improvement of cardiac function after myocardial infarction (15).

It was hypothesized that glomerular endothelial cells, which undergo phenotypic changes to a proangiogenic type, are the culprit of glomerular filtration barrier leakage in diabetic nephropathy. The study was designed to determine the role of glycolysis in proangiogenic glomerular endothelial cells in diabetes and the signaling cascade involved in vivo and in vitro.

## METHODS

### Animal Experiments

Male and female type 2 diabetic mice, *db/db* mice, and their genetic control mice were bought from Cavens Laboratory Animal Corporation (Zhejiang, China). Mice were housed in cages under clean and temperature-controlled conditions in the Laboratory Animal Unit of Zhongshan Hospital (Shanghai, PRC). The mice were fed regular chow and given free access to water. Mice were euthanized with 1.2 g/kg urethane (Sigma-Aldrich) by intraperitoneal injection when they were 10 wk old. Blood samples, renal cortex tissue, heart, and lung were collected. The animal study was approved by the Zhongshan Hospital animal care committee (No. ZS2017-015) and was carried out in accordance with the UK Animals Act, 1986, and associated guidelines. Data collection and evaluation of all experiments were performed blindly of the group identity.

### MicroRNA Delivery In Vivo and In Vitro

Since diabetic nephropathy is a chronic disease, long-term treatment is required. Delivery of miRNA agomir in mouse kidneys was examined in three approaches when mice were 7 wk old. Systemic administration via tail vein injection, every week for three times, increased the miRNA expression in the lung, but not the kidney (15 µg prepared with lipofectamine RNAiMAX in 100 µL DMEM medium, Supplemental Fig. S1). It is also reported that local administration of miRNA maintains a high level in the hearts for at least 4 wk (15). In the present study, intrarenal injection increased serum creatinine levels (one shot of 15 µg prepared with lipofectamine, data not shown). Then, the administration of miRNA via perirenal adipose tissue was optimized and used in the present study. In brief, mice were induced anesthesia with pentobarbitone (50 mg/kg ip, Sigma-Aldrich), and then the left kidney was carefully exposed. A total of 15 µg agomir or negative control (NC) (prepared with lipofectamine in 200 µL medium; miRNA agomir: *n* = 11, negative agomir: *n* = 5) was injected into the perirenal adipose tissue of the kidney. Three weeks later, mice were euthanized for sample collection (Supplemental Fig. S2).

To overexpress miR-590-3p in cultured cells, human glomerular endothelial cells were transfected with has-miR-590-3p mimic (50 ng/mL, RiboBio, Guangzhou, China) and its negative control (NC), using Lipofectamine RNAiMAX (Invitrogen, Waltham, MA). Silencing PFKFB3 plasmids were delivered by RNAiMAX (sense 5′-GAG CCU GUG AUC AUG GAA UTT-3′, antisense 5′-AUU CCA UGA UCA CAG GCU CTT-3′).

### Renal Cells Preparation for Single-Cell Sequencing

Renal cortex of control mice, 6-wk-old *db/db* mice (high serum glucose levels without organ damage), and 10-wk-old *db/db* mice [shown diabetic complications, including cognitive dysfunction (20) and albuminuria] were collected and digested in Hanks buffer with 10 mg/mL of collagenase IV (Sigma-Aldrich) and 200 U/mL of DNase I (Invitrogen, Carlsbad, MO) at 37°C for 20 min. Renal cortex samples were dissociated using a gentle MACS Dissociation (Miltenyi Biotec, Bergisch Gladbach, Germany) according to the manufacturer’s instructions (21).

### Single-Cell cDNA and Library Preparation

Chromium Single Cell 3′ Library & Gel Bead Kit v2 (PN-120237), Chromium Single Cell 3′ Chip kit v2 (PN-120236), and Chromium i7 Multiplex Kit (PN-120262) were used for 10× Genomics examination according to the manufacturer’s instructions. Single-cell suspensions were prepared with PBS containing 0.04% bovine serum albumin. Cell viability and counts were verified with a hemocytometer. Approximately 14,000 cells were subjected to the 10× Genomics Chromium Controller machine for Gel Beads-in-Emulsion (GEM) generation. Messenger RNA was prepared using 10× Genomics Chromium Single Cell 3′ reagent kit (V2 chemistry), while cells were partitioned into the GEMs with cell-specific and transcript-specific barcodes [16 bp 10× Barcode and 10 bp Unique Molecular Identifier (UMI), respectively]. After RT-PCR, cDNA was recovered, purified, and amplified for library preparation.

### 3′-End Single-Cell RNA-Sequencing and Data Processing

Agilent Bioanalyzer2100 was used for library quality control and concentration measurement. Libraries were run on the Hiseq Xten for Illumina PE150 (Illumina, San Diego, CA). Postprocessing and quality control were performed by using the 10× Cell Ranger package (v.3.1.0, 10× Genomics). The sequencing reads were examined by quality metrics, and transcripts were mapped to a reference mouse genome (mm10) and assigned to individual cells of origin according to the cell-specific barcodes using the Cell Ranger pipeline (10× Genomics). Single UMIs were counted for gene expression level analysis and downstream analyses.

### Single-Cell Sequencing Data Sources and Analyses

The online datasets (GSE127235) were generated in glomerular cells of streptozotocin-induced type 1 diabetic mice on the endothelial nitric oxide synthase (eNOS)-deficient background (5).

R software and Seurat package (v.3.1.2) were used to process the single-cell sequencing data of *db/db* mice and streptozotocin-induced type 1 diabetic mice (22, 23). After gene name conversion, the Seurat package with min.cells = 3 and min.features = 200 was used. To exclude data of doublet and multiplets, gene counts between 200 and 3,500 and the mitochondrial gene percentage less than 30% were set as the criteria. Run Principal Component Analysis was set with npcs = 40. Uniform manifold approximation and projection (UMAP) presented clusters of cells with dims = 1:40 and reduction = “pca.” T-distributed stochastic neighbor embedding was generated by RUNTSNE with dims = 1:40 and reduction = “pca.” FindNeighbors was set with dims = 1:40. Clusters of cells were identified with the FindClusters with resolution = 0.8. Differentially expressed genes (DEGs) were detected with FindAllMarkers function by Wilcox rank-sum test when log-fold change.threshold = 0.25, min.pct = 0.1. Highly expressed genes were identified by an adjusted *P* value less than 0.05 and a false discovery rate less than 0.05. Cell type assignment was performed based on previous studies (5, 24) and CellMaker database (25).

By using the FindMarkers function with the Wilcox test, DEGs were defined when absolute foldchange was higher than 1.5 or lower than 0.67 with a *P* value less than 0.05. ClueGO (26), a plug-in in Cytoscape 3.8.3(RRID:SCR_003032), was used to analyze gene ontology and DEGs enrichment. Function clusters were calculated using kappa-score on their biological roles and presented in pie charts.

### Glomerular Single Cells Preparation for SMART RNA-Seq and Analyses

Mouse glomeruli were collected by Dynabeads and a magnetic particle concentrator (Invitrogen, Carlsbad, MO). After perfusion of ice-cold PBS with 8 × 10^7^ Dynabead ferric beans M450 (Invitrogen, Carlsbad, MO) for 20 min (2 mL/min), mouse kidneys were digested with collagenase A (Roche, Basel, Switzerland) at 37°C for 30 min and filtered with 100-µm cell strainers. The glomeruli, collected by a magnetic particle concentrator (Invitrogen, Carlsbad, MO), were further incubated with Hanks buffer with 10 mg/mL of collagenase IV (Sigma-Aldrich) and 200 U/mL of DNase I (Invitrogen, Carlsbad, MO) at 37°C for 20 min (21). Glomerular cells, stained for lineage markers CD31 (endothelial cells, 1:100, 551262, RRID:AB_398497, BD PharMingen), Podoplanin (podocytes, 1:100, 566390, RRID:AB_2739721, BD PharMingen), and CD90 (mesangial cells, 1:100, 561973, RRID:AB_10898011, BD PharMingen) were sorted using a BD AriaIII (BD Bioscience, Switzerland).

Mouse orthologs of human genes were obtained using the biomaRt R package (27). DEseq2 (RRID:SCR_015687) (28) was used to identify the DEGs for glomerular endothelial cells of 6-wk and 10-wk diabetic mice. In the present study, Wilcox test, a nonparametric test for a single population centroid was used. DEGs were defined when a gene with fold changes higher than 2 and *P* value less than 0.05.

### Endothelial Cell Culture

Primary human renal glomerular endothelial cells (HGECs, Cat. No. 4000, Lot No. 20485) were purchased from ScienCell (San Diego, CA). Endothelial cells were cultured and passaged (from *passage 4* to *6*) in endothelial culture medium (ScienCell, 5.5 mM glucose). After 16-h starvation (1% fetal bovine serum), experiments were performed in DMEM medium with 5.5 mM glucose. In the present study, cultured GECs were challenged with 22.2 mM glucose (16.7 mM glucose added in medium, 16.7 mM mannitol added as an osmotic control) in the presence and absence of a sprouting regimen containing vascular endothelial growth factor A (VEGFA, 50 ng/mL; Peprotech), sphingosine 1-phosphate (S1P, 1 µM; Sigma-Aldrich), and phorbol 12-myristate 13-acetate (PMA, 2 ng/mL; Sigma-Aldrich) (29). After stimulations, cells were harvested for Western blotting and real-time polymerase chain reaction. 2-Deoxy-d-glucose (2-DG, 20 mM) was added to the medium 2 h before the sprouting regimen and high glucose stimulation. Deta-NONOate (DetaNO, 1 mM), *N*_ω_-nitro-l-arginine-methyl ester (l-NAME, 0.1 mM), SEW2874 (30 nM), and FTY720 (100 nM) were added to the medium 1 h before the sprouting regimen and high glucose stimulation.

### RNA Extraction and Quantitative Real-Time Polymerase Chain Reaction

Total RNA was extracted from cultured GECs by TRIzol reagent (Takara, Otsu, Japan) according to the manufacturer’s instructions. Expression levels of miRNAs were assessed by qRT-PCR using Bulge-Loop TM miRNA qRT-PCR Starter Kit (RiboBio, Guangzhou, China) on ABI 7500 System. The Bulge-LoopTM RT-qPCR primers for miR-590-3p and U6 small nuclear RNA were from RiboBio Company. For mRNAs analysis, cDNA was synthesized with high-capacity cDNA reverse transcription kit (Takara). Quantitative real-time polymerase chain reaction (RT-PCR) was performed with SYBR green qPCR master mix (Takara). The results of real-time PCR were quantified using the 2^−ΔΔCt^ method (Table 1).

**Table 1.** Primer information

Gene	Forward	Reverse
*Angpt1*	CCCTAAGCCATCAGCAATCCTT	GTTGCACATCCAAGCCAAGC
*Angpt2*	TTGAACCAAACAGCGGAGCA	TGTCGAGAGGGAGTGTTCCA
*Claudin1*	TGAGGATGGCTGTCATTGGG	AAAGTAGGGCACCTCCCAGA
*Claudin5*	CTCTGCTGGTTCGCCAACAT	CAGCTCGTACTTCTGCGACA
*Claudin11*	CTTGGCACTGTAGCATGTGGA	GACCGAGGCAGCAATCATCA
*GAPDH*	TTGGTATCGTGGAAGGACTCA	CCAGTGAGCTTCCCGTTCAG
*Glut1*	CTTTGTGGCCTTCTTTGAAGT	CCACACAGTTGCTCCACAT
*Glut4*	TGGAAGGAAAAGGGCCATGCTG	CAATGAGGAATCGTCCAAGGATG
*HK2*	GATTGTCCGTAACATTCTCATCGA	TGTCTTGAGCCGCTCTGAGAT
*PDE3A*	GGGGGCCACTGGGAATTCAG	GACTCACCCCACTGTGGACG
*PFKFB3*	AGCCCGGATTACAAAGACTGC	GGTAGCTGGCTTCATAGCAAC
*PKM2*	CTATCCTCTGGAGGCTGTGC	CCATGAGGTCTGTGGAGTGA
*S1PR1*	CGACTCGAGCTGCGGTTT	GCTACTCCAGACGAACGCTA
*Tubulin*	TGGACTCTGTTCGCTCAGGT	TGCCTCCTTCCGTACCACAT
*VCAM1*	CATGTAGTGTCATGGGCTGTG	CTCAGGGTCAGCGTGGAAT
*VEGFA*	ACGAAAGCGCAAGAAATCCC	GGAGGCTCCAGGGCATTAG

HK2, hexokinase 2; PDE3A, phosphodiesterase 3A; PFKFB3, phosphofructokinase/fructose bisphosphatase 3; PKM2, pyruvate kinase enzyme M2; S1PR1, receptor for sphingosine 1-phosphate; VEGFA, vascular endothelial growth factor A.

### Western Blotting

Protein presences were determined by Western blotting. Cultured GECs were prepared in lysis buffer containing phenyl ethyl malonylurea fluoride and 1% NP-40 (Cell Signaling, Danvers, MA). Protein concentrations were measured using the Bradford assay (Bio-Rad, Hercules, CA). A total of 20 µg protein samples were separated by SDS-PAGE and transferred to polyvinylidene fluoride membranes (Millipore, Temecula, CA). Parallel blots from the same experiment were processed when target proteins were difficult to be separated by SDS-PAGE gel. Blots of total eNOS and protein kinase A (PKA) were examined after striping their phosphorylated forms. Related signals were normalized to α-tubulin and quantified using Scion Image software (Scion Corp, Frederick, MD, RRID:SCR_008673).

### Luciferase Assay

The luciferase assay was performed according to the manufacturer’s instructions (No. RG207 Bioyotime Biotech Shanghai, China). In brief, 25 ng/mL of plasmid were transfected with the duplex of has-miR-590-3p mimic and Lipofectamine RNAiMAX.

### Glycated Rate Assay

The glycated rate in cultured glomerular endothelial cells was measured by Agilent Seahorse XF glycated rate assay kit (Agilent Tech, Wilmington, DE). A total of 1 × 10^6^ GECs were seeded in an Agilent 96-well plate and stimulated with high glucose in the presence and absence of the sprouting regime. The glycated rate measurement was performed 6 h after the stimulation.

### Cell Viability Test

Cell viability was examined by MTT (3-[4,5-dimethylthiazol-2-yl]-2,5 diphenyl tetrazolium bromide) test according to the manufacturer’s instructions (No. KGA 321 Keygen Biotech Jiangsu, China).

### TUNEL Test

Cell apoptosis was examined by the TUNEL test (C1089 Beyotime Biotechnology, Shanghai, China). Images were captured with a color video camera (DP71; Olympus, Tokyo, Japan) connected to a microscope (DPBX51; Olympus). The TUNEL signals were examined by a blinded technician and analyzed by Scion Image software (ImageJ, Scion Corp, Frederick, MD, RRID:SCR_003070).

### Cell Migration Test

A total of 3 × 10^5^ endothelial cells were seeded in 6-well sterile culture plates. GECs were stimulated with high glucose in the presence and absence of the sprouting regimen. To avoid the sprouting regimen-induced responses, a scratch was created by a P1000 pipette tip 6-h after the stimulation. Cell migration was recorded for 42 h (microscope DPBX51; Olympus) and examined by a blinded technician using Scion Image software (Scion Corp, Frederick, MD, RRID:SCR_008673).

### Assessment of Angiogenic Potential in Matrigel

The angiogenic potential assessment was performed on Matrigel (No. 354230, Invitrogen, Carlsbad, MO). GECs (3 × 10^4^) were seeded in a 96-well plate and incubated with the sprouting regimen. Since GECs failed to form typical tubes within 24 h, prolonged observation was performed for 30 days. Filopodia and tube formation were visualized with Calcein acetoxymethyl ester (PK-CA707-80011, PromoCell, Tokyo, Japan) under fluorescence microscopy (DPBX51; Olympus). The filopodia length was examined by a blinded technician and analyzed by Scion Image software (Scion Corp, Frederick, MD, RRID:SCR_008673).

### Hematoxylin & Eosin Staining and Immunohistochemistry

Mouse kidneys were fixed with a 10% formalin solution for at least 24 h and embedded in paraffin. Antigen retrieval was carried out by incubating the samples with 0.3% H_2_O_2_ for 30 min and heating to boiling with microwaves in the citrate buffer for 10 min. The sections were blocked with 5% goat serum in Tris-buffered saline and then incubated overnight at 4°C with diluted primary antibodies. The sections were further incubated in Super-Picture 3rd Gen IHC detection kits (Invitrogen, Carlsbad, MO). Sections without primary antibodies were used for negative control staining (30).

To quantify positive signals in glomeruli, images with a magnification of ×400 were used. At least 20 glomeruli were analyzed from each kidney sample using the ImageJ software (RRID:SCR_003070). First, the images were converted to grayscale to set up the threshold levels for staining detection. Then, images were converted to binary (black and white) followed by region of interest (ROI) measurement using the freehand tool. To determine the area stained within the ROI, selecting Analyze/Analyze Particles revealed a summary that included the area fraction for the proportion of the ROI with positive staining.

### Transmission Electron Microscope

The renal cortex was fixed with 4% paraformaldehyde and prepared by the Chinese Academy of Science.

### Creatinine Measurement

Plasma and urine samples were obtained by centrifugation (500 *g*) for 5 min at 4°C and stored at −20°C. Creatinine levels were measured using the Quantichrom Creatinine assay kit (BioAssay System; Hayward, CA).

### Urine Albumin Measurement

Urine levels of albumin were measured using a BCP albumin assay kit, according to the manufacturer’s instructions (Sigma-Aldrich, St. Louis, MO).

### Reagents

Mimics of miR-590-3p were brought from Ribobio Tech (RiboBio, Guangzhou, China). Glucose, mannitol, PMA, S1P, and collagenase IV were bought from Sigma-Aldrich (St. Louis, MO). VEGFA was brought from Peprotech (Rocky Hill, NJ). Fingolimod and SEW2871 were obtained from Medchemexpress (MCE, Shanghai, China). DNase I was purchased from Invitrogen (Invitrogen, Carlsbad, MO). Anti-Bcl-2 (Cat. No. 2876, 1:1,000 WB, RRID:AB_2064177), caspase-3 (Cat. No. 9665, 1:1,000 WB, RRID:AB_2069872), cleaved caspase-3 (Cat. No. 9661, 1:1,000 WB RRID:AB_2341188), PFKFB3 (Cat. No. 13123, 1:1,000 WB, RRID:AB_2617178), PKA (Cat. No. 5842, 1:1,000 WB, RRID:AB_10706172), phosphor-PKA (Cat. No. 5661, 1:1,000 WB, RRID:AB_10707163), VEGFR2 (Cat. No. 9698, 1:1,000 WB, RRID:AB_11178792), and phosphor-VEGFR2 (Cat. No. 2478, 1:1,000 WB, RRID:AB_331377) antibodies were purchased from Cell Signaling (Danvers, MA). Anti-Claudin 1 (Cat. No. ab15098, 1:1,000 WB, 1:200 IHC, RRID:AB_301644), Claudin 5 (Cat. No. ab15106, 1:1,000 WB, 1:200 IHC, RRID:AB_301652), VEGFR1 (Cat. No. ab32152, 1:1,000 WB, RRID:AB_778798), VEGFR3 (Cat. No. ab27278, 1:1,000 WB, RRID:AB_470949), S1pR1 (Cat. No. ab77076, 1:1,000 WB, 1:100 IHC, RRID:AB_1523525) and, VEGFA (Cat. No. ab1316, 1:200 IHC, RRID:AB_299738) antibodies were purchased from Abcam (Cambridge, UK). Anti-angiopoietin2 (Cat. No. NB110-85467, 1:1,000 for WB, 1:200 for IHC, RRID:AB_1199462), and phosphodiesterase 3A (PDE3A, Cat. No. NBP1-46182, 1:1,000 WB, RRID:AB_10009414) antibodies were purchased from Novus (Centennial, CO). Antiangiopoietin 1 (Cat. No. AF923, 1:1,000 WB, 1:100 IHC, RRID:AB_355715) was purchased from the R&D system (Minneapolis, MN). Anti-eNOS (Cat. No. 610296, 1:2,000 WB, 1:200 IHC, RRID:AB_397690) and phosphor-eNOS (Cat. No. 612393, 1:2,000 for WB, RRID:AB_399751) antibodies were purchased from BD Transduction Laboratories (NJ). Muscle-associated glycogen phosphorylase (PYGM; Cat. No. 19716-1-AP, 1:1,000 WB, RRID:AB_10642009), CX3CL1 (Cat. No. 10108-2-AP, 1:200 IHC, RRID:AB_2087154), and PFKFB3 (Cat. No. 13763-1-AP, 1:200 for IHC, RRID:AB_2162854) antibodies were purchased from Proteintech (Hubei, China). α-Tub (Cat. No. 39527, 1:5,000 WB, RRID:AB_2793243) antibody was purchased from Active Motif (Carlsbad, CA). Anti-rabbit (Cat. No. 115-035-003, 1:2,000 WB, RRID: AB_2313567) and anti-mouse (Cat. No. 111-035-003, 1:2,000 WB, RRID:AB_10015289) secondary antibodies were purchased from Jackson ImmunoResearch Laboratories (West Grove, PA).

### Data Analysis

Data are presented as means ± SE; *n* refers to the number of mice or the number of individual experiments in cell culture. Unpaired *t* test and one-way ANOVA by Bonferroni post hoc test were performed (GraphPad Prism, v.5, GraphPad Software, San Diego, CA, RRID:SCR_002798). Differences were considered to be statistically significant when *P* was less than 0.05.

## RESULTS

### A Subgroup of Glomerular Endothelial Cells Possesses Angiogenic Features in the Kidney of Diabetic Mice

**Figure 1.**
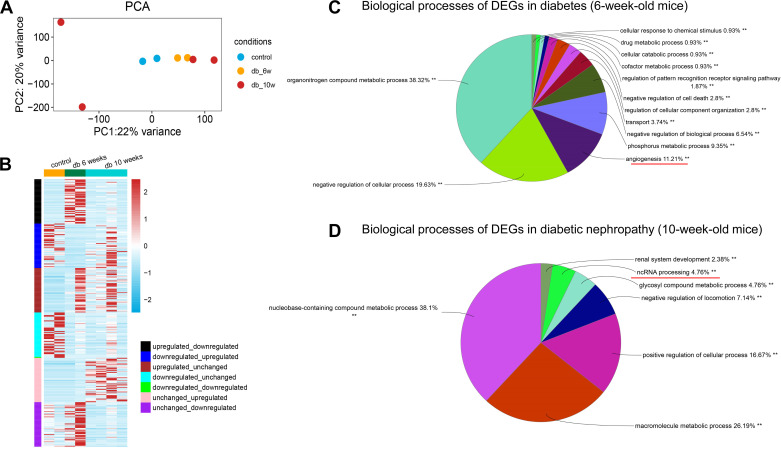
Glomerular endothelial cells in mice with diabetic nephropathy exhibit great heterogeneity. *A*: isolated glomerular endothelial cells (GECs) in principle component analysis. *B*: heatmap of top 50 differentially expressed genes (DEGs) in the development of diabetic nephropathy. *C*: pie chart of significantly enriched gene ontology (GO) terms group from DEGs in the comparison of 6-wk *db/db* and control mice. *D*: pie chart of significantly enriched GO terms group in the comparison of mice of diabetes (6 wk old) and diabetic nephropathy (10 wk old).

In isolated glomerular endothelial cells, RNA-sequencing reported heterogeneity in 10-wk diabetic mice in principle component analyses (Fig. 1*A*). By pooling DEGs that were transiently increased or decreased in 6-wk-old diabetic mice (Fig. 1*B*), the enrichment of DEGs was observed in the biological processes of angiogenesis, regulation of cell death, and changes in metabolic processes (Fig. 1*C*). In glomerular endothelial cells of 10-wk-old diabetic mice, DEGs were involved in renal system development and negative regulation of locomotion, and noncode RNA processing (Fig. 1*D*). It implies that angiogenesis occurs in the early stage of diabetes and is followed by renal system development. Of note, noncode RNA processing plays a role in the progress of diabetic nephropathy.

**Figure 2.**
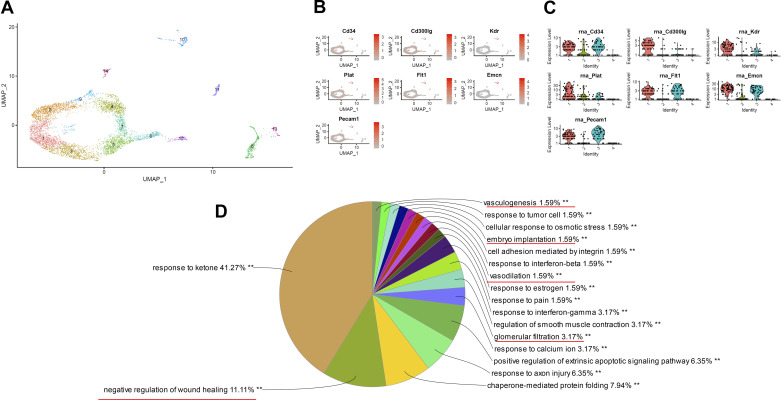
Type 2 diabetic *db/db* mice have a subgroup of glomerular endothelial cells (GECs) processing proangiogenic characteristics. *A*: uniform manifold approximation and projection (UMAP) plot of renal cortex cells in type 2 diabetic mice. *B*: representative endothelial cell markers in *Cluster 10* in UMAP. *C*: violin plot showing representative endothelial cell markers in *Cluster 10*. *D*: pie chart of significantly enriched GO terms group from differentially expressed genes (DEGs) in the comparison of two subclusters of GECs in *Cluster 10*.

To further confirm the progress of angiogenesis in diabetic nephropathy, single-cell sequencing was conducted in diabetic mice as well. There were 5,976 cells found in the kidneys of diabetic mice, in which 4,721 cells were proximal tubule cells. UMAP revealed that 115 endothelial cells (1.9%) (4, 21, 24), 46 mesangial cells, and 20 macrophages were in *cluster 10* (Fig. 2, *A* and *B*). Endothelial cells with high expressions of Emcn, Kdr, Flt1, and Pecam1 were further subdivided into two clusters (Fig. 2*C*), with 68 cells in *subcluster 0* (control:diabetes6w:diabetes10w =19:14:35) and 47 cells in *subcluster 2* (control:diabetes6w:diabetes10w = 12:3:32). Compared with endothelial cells in *subcluster 0*, DEGs in *subcluster 2* were enriched in modules of negative response to wounding, response to axon injury, glomerular filtration, positive regulation of extrinsic apoptotic signaling pathway, vasodilatation, embryo implantation, and vasculogenesis (Fig. 2*D*). Consistent with the aforementioned noncode RNA in RNA sequencing, gene silencing by miRNA is a critical player in the development of diabetic nephropathy (Supplemental Fig. S3*D*).

Together with the *s*ingle-cell sequencing data from patients with diabetes (6) and type 1 diabetic mice (Supplemental Figs. S4 and S5), the present results supported the claim that a subgroup of glomerular endothelial cells exhibiting angiogenic features in the development of diabetic nephropathy (4–6). Therefore, further experiments were designed to investigate the role of proangiogenic glomerular endothelial cells in the disease.

### Expressions of miRNA-590-3p in Glomerular Endothelial Cells Challenged with High Glucose

**Figure 3.**
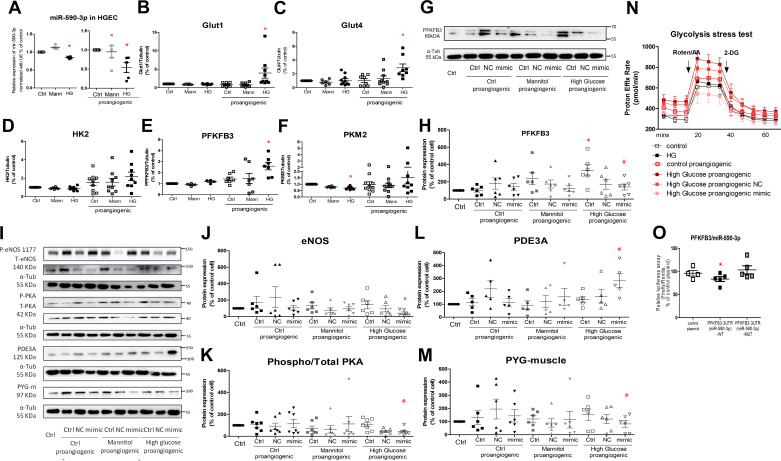
*A*: microRNA-590-3p expressions in control and proangiogenic glomerular endothelial cells (GECs) incubated without (Ctrl) or with high glucose (HG) or mannitol (Mann), the osmatic control of high glucose. Messenger RNA expressions of the rate-limiting enzymes Glut1 (*B*), Glut4 (*C*), hexokinase 2 (HK2, *D*), phosphofructokinase/fructose bisphosphatase 3 (PFKFB3, *E*), pyruvate kinase enzyme M2 (PKM2, *F*) in glycolysis in GECs in the presence (the proangiogenic cells) or the absence of the sprouting regimen. Representative Western blots (*G*) and densitometric quantification of PFKFB3 in proangiogenic GECs (*H*), transfected without (ctrl) or with the negative control miRNA (NC) or miRNA-590-3p mimic (mimic), after 6-h stimulation; *n* = 6, *P < 0.05 compared with the cells under control (without stimulation), #*P* < 0.05 compared with control cells stimulated with high glucose and sprouting regimen using one-way ANOVA. *I*: representative Western blots of nitric oxide synthase, endothelial (eNOS), protein kinase A (PKA), phosphodiesterase 3A (PDE3A), and muscle-associated glycogen phosphorylase (PYGM) protein. Densitometric quantification of total eNOS (*J*), phosphorylated PKA (*K*), PDE3A (*L*), and PYGM (*M*) protein in proangiogenic GECs. *n* = 5 or 6, #*P* < 0.05 compared with human renal glomerular endothelial cell (HGEC) stimulated with high glucose and sprouting regimen using one-way ANOVA. *N*: glycated rate measured by seahorse influx analyses in proangiogenic GECs, transfected without or with miRNA-590-3p mimic. *n* = 6. *O*: regulation of PFKFB3 by miRNA-590-3p in Luciferase assay. *n* = 4. *P < 0.05 compared with the cells under control using one-way ANOVA.

In the present study, high glucose significantly reduced miRNA-590-3p expression in cultured GECs in the presence and absence of a sprouting regimen containing VEGFA, S1P, and PMA (29) (Fig. 3*A*).

### High Glucose Enhances Glycolysis in Proangiogenic GECs but Not in Quiescent GECs

Since glycolysis is the principle energy setting for endothelial cells (7, 8), expressions of the rate-limiting glycolytic enzymes in GECs were examined in the presence (proangiogenic) and absence of the sprouting regimen. High-glucose stimulation reduced PKM2 mRNA expression, but not the other rate-limiting enzymes in GECs in the absence of the sprouting regimen (Fig. 3, *B–F*), whereas high-glucose incubation significantly increased mRNA expression of GLUT1, GLUT4, and PFKFB3, but had no effects on HK2 or PKM2 in GECs stimulated with the sprouting regimen (Fig. 3, *B–F*). Incubation of mannitol, the osmotic control, did not significantly change mRNA expressions of the glycolytic enzymes.

Both HK2 and PFKFB3 were predicted as miRNA-590-3p targets by the online tools, miRNA-590-3p mimic was transfected in GECs challenged with high glucose and the sprouting regimen. Mimic of miRNA-590-3p significantly reduced high-glucose-induced PFKFB3 (Fig. 3, *G* and *H*), but not HK2 expressions (Supplemental Fig. S6). Consistently, PFKFB3 protein level was not changed in HGECs incubated with mannitol and sprouting regimen (Fig. 3, *G* and *H*).

In addition to the reduced expression of PFKFB3 by miRNA-590-3p mimics-transfection, miRNA-590-3p mimic also reduced protein expressions of glycogenolysis enzyme, PYGM, in the GECs challenged with high glucose and the sprouting regimen (Fig. 3, *I* and *M*). MicroRNA-590-3p mimic significantly reduced eNOS total protein expression, as well as phosphorylated Ser1177 of eNOS, and the phosphorylation of PKA, but increased PDE3A protein expression in GECs challenged with high glucose and the sprouting regimen (Fig. 3, *I–L*).

Likewise, seahorse influx analysis revealed that high glucose or the sprouting regimen alone did not impact the glycated rate in GECs. The combined stimulation significantly increased endothelial glycolysis, which was prevented by miRNA-590-3p overexpression (Fig. 3*N*).

Consistent with predictions from TargetScan, luciferase assay reported that endothelial cells transfected with wild-type, but not mutant, plasmids of PFKFB3 had fewer luminant signals than those transfected with their negative control plasmid (Fig. 3*O*).

### Inhibition of Glycolysis in Proangiogenic Endothelial Cells Increases Angiogenesis Regulator S1pR1 Expression

**Figure 4.**
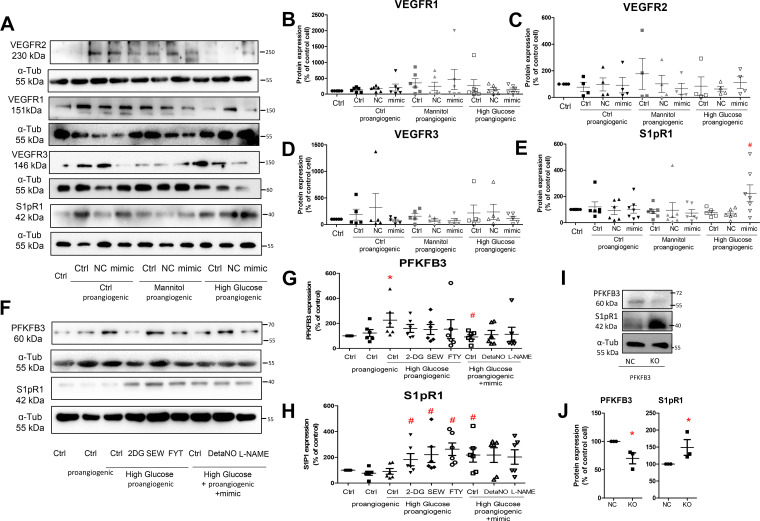
*A*: representative Western blots of vascular endothelial growth factor receptor (VEGFR)1, VEGFR2, VEGFR3, and receptor for sphingosine 1-phosphate (S1pR1) in proangiogenic glomerular endothelial cells (GECs). Densitometric quantification of VEGFR1 (*B*), VEGFR2 (*C*), VEGFR3 (*D*), and S1pR1 (*E*) in proangiogenic GECs. *n* = 6. #*P* < 0.05 compared with human renal glomerular endothelial cell (HGEC) stimulated with high glucose and sprouting regimen using one-way ANOVA. *F*: representative Western blots of PFKFB3 and S1pR1 protein and densitometric quantification of PFKFB3 (*G*) and S1pR1 (*H*). *n* = 6, *P < 0.05 compared with the cells under control (without stimulation), #*P* < 0.05 compared with high glucose and sprouting regimen using one-way ANOVA and PFKFB3 (*H*) protein in proangiogenic GECs after high glucose stimulation. *n* = 6, *P < 0.05 compared with control cells, #*P* < 0.05 compared with cells stimulated with high glucose and sprouting regimen using one-way ANOVA. Genetic deletion of PFKFB3 (KO), compared with its negative control (NC), increases S1pR1 protein expression in proangiogenic GECs stimulated with high glucose. Representative Western blots of PFKFB3 and S1pR1 (*I*) and densitometric quantification of PFKFB3 and S1pR1 protein (*J*) in proangiogenic GECs stimulated with high glucose. *n* = 3, *P < 0.05 compared with the NC cells using Student *t* test.

Angiogenesis regulators include angiopoietins (ANGPT1 and ANGPT2) (31, 32), VEGF-VEGFRs (33, 34), and S1pR1 (35–37). In the present study, glucose or the sprouting regimen did not change the mRNA expressions of angiopoietins or VEGFA in GECs (Supplemental Fig. S7). High glucose, the sprouting regimen, or the combination also did not change the protein expressions of VEGFRs, including VEGFR1, VEGFR2, or VEGFR3 (Fig. 4, *A–D*). In proangiogenic GECs, high glucose alone did not affect S1pR1 protein expression. Treatment of miRNA-590-3p mimics significantly increased S1pR1 protein expressions in the proangiogenic GECs challenged with high glucose (Fig. 4, *A* and *E*), but not with mannitol. The involvement of S1pR2 in angiogenesis has been reported in the endothelial cells of the blood-brain barrier (38) and in mouse cardiac microvascular endothelial cells in vitro experiments (39). However, the presence of S1pR2 was not detected in cultured GECs in the present study.

Neither nitric oxide donor DetaNO, nor nitric oxide synthase inhibitor l-NAME, significantly altered PFKFB3 and S1pR1 protein expression in proangiogenic GECs transfected with miRNA-590-3p mimics (Fig. 4, *F–H*). FTY720, a sphingosine-1-phosphate receptor modulator, and SEW2871, a highly selective S1pR1 agonist, increased S1pR1 protein expression in cultured GECs, but did not affect the PFKFB3 protein expression (Fig. 4, *F–H*).

To study the role of glycolysis in regulating S1pR1 expression, 2-DG, a pharmacologic inhibitor of glycolysis, and genetic deleting PFKFB3 were used in the presence of high glucose and the sprouting regimen. Incubation of 2-DG increased S1pR1 protein expression in GECs compared with control cells (Fig. 4, *F* and *H*). Deleting PFKFB3 increased S1pR1 protein expression in GECs (Fig. 4, *I* and *J*).

### High Glucose Increases Apoptosis in Control GECs, but Not in Proangiogenic GECs

**Figure 5.**
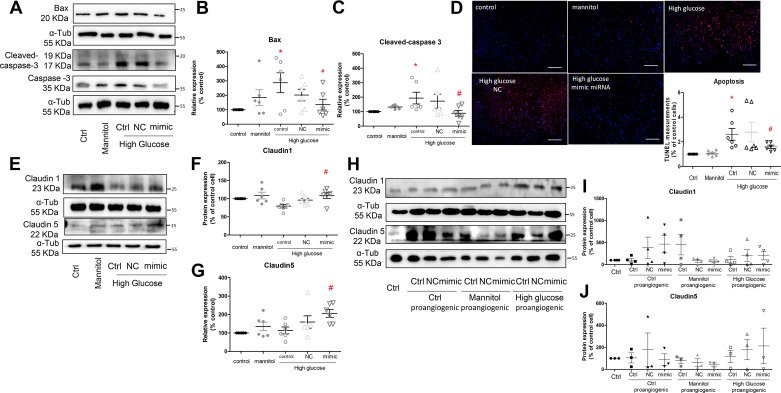
*A*: representative Western blots of Bax, cleaved-caspase 3, and caspase 3 in quiescent glomerular endothelial cells (GECs), transfected without or with the negative control (NC) or miRNA-590-3p mimic (mimic) stimulated with high glucose, and densitometric quantification of Bax (*B*) and cleaved-caspase 3 (*C*) in quiescent GECs. *n* = 6, *P < 0.05 compared with cells under the control condition. #*P* < 0.05 compared with cells stimulated with high glucose using one-way ANOVA. *D*: fluorescent signals of TUNEL in quiescent GECs transfected without or with miRNA-590-3p mimic. Scale bar: 100 µm. *E*: representative Western blots of Claudin 1 and Claudin 5 in quiescent GECs, transfected without or with miRNA-590-3p mimic, and densitometric quantification of Claudin 1 (*F*) and Claudin 5 (*G*) in quiescent GECs. *n* = 6, #*P* < 0.05 compared with cells stimulated with high glucose using one-way ANOVA. *H*: representative Western blots of Claudin 1 and Claudin 5 in proangiogenic GECs, transfected without or with miRNA-590-3p mimic, and densitometric quantification of Claudin 1 (*I*) and Claudin 5 (*J*) in proangiogenic GECs, *n* = 3 or 4.

High glucose significantly increased apoptotic protein expressions of cleaved-caspase 3 and Bax in quiescent GECs (Fig. 5, *A–C*). Treatment of miRNA-590-3p mimics significantly reduced the protein expressions (Fig. 5, *A–C*). In line with the upregulation of apoptotic proteins, high glucose increased the TUNEL signals in quiescent GECs but not in those transfected with miRNA-590-3p mimics (Fig. 5*D*).

Transfection with miRNA-590-3p mimics increased Claudin 1 and Claudin 5 protein expressions in quiescent GECs challenged with high glucose (Fig. 5, *E–G*), but not in proangiogenic GECs (Fig. 5, *H–J*).

### MicroRNA-590-3p Reduces Endothelial Migration and Impairs Filopodia in Proangiogenic GECs

**Figure 6.**
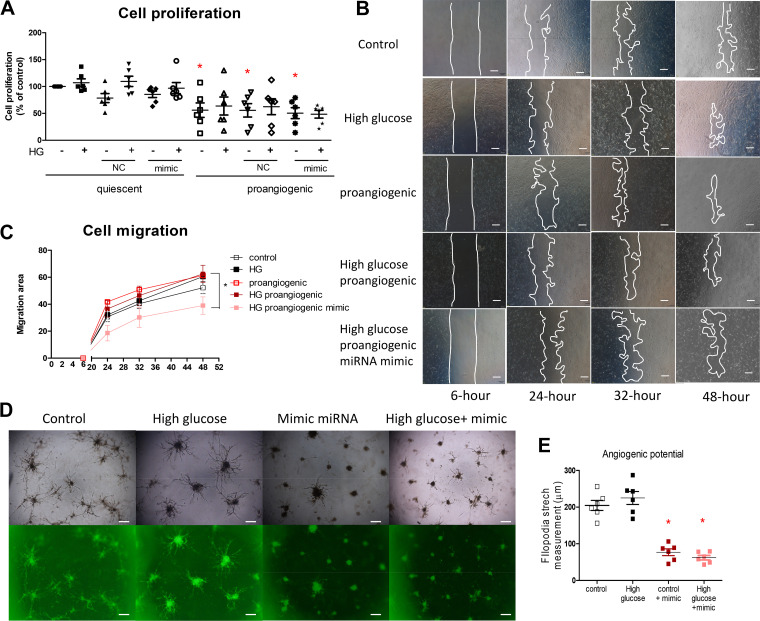
*A*: cell proliferation in MTT assay. *n* = 6, *P < 0.05 compared with the respective quiescent cells using one-way ANOVA. *B*: representative pictures, scale bar: 100 µm, and quantification of cell migration (*C*) in glomerular endothelial cells (GECs) transfected without or with miRNA-590-3p, stimulated with high glucose and/or sprouting regimen. *n* = 6, *P < 0.05 using one-way ANOVA. *D*: representative image of angiogenesis, scale bar: 100 µm, in bright-field (*top*) and visualized with calcein acetoxymethyl under fluorescence microscopy (*bottom*) and quantification of filopodia stretches in Matrigel (*E*). *P < 0.05 compared with the respective group without transfecting with miRNA-590-3p mimic using one-way ANOVA. NC, negative control; HG, high glucose.

The sprouting regimen slightly reduced GECs viability, which was not affected by high-glucose incubation. Combined incubation of high glucose and miRNA-590-3p mimics did not have additional effects (Fig. 6*A*).

In the scratch experiment, high glucose did not affect GECs migration in either control or proangiogenic conditions. Overexpressing miRNA-590-3p reduced endothelial migration when proangiogenic GECs were exposed to high glucose (Fig. 6, *B* and *C*).

Unlike umbilical and aortic endothelial cells (Supplemental Fig. S8), cultured GECs failed to form typical tubes (Fig. 6*D*). In response to the sprouting regimen, GECs aggregated, and some filopodia stretched out. The spheroids extended their filopodia for 30 days, and some filopodia were connected (Fig. 6*D* and Supplemental Fig. S9). In the presence of high glucose, fewer connections were observed, but filopodia sprouting and stretching were not affected in endothelial cells. Overexpressing miRNA-590-3p, either in the control condition or exposed to high glucose, had shorter filopodia (Fig. 6, *D* and *E*).

### Perirenal Adipose Tissue Delivery of miRNA-590-3p Restores Glomerular Endothelial Function and Reduces Albuminuria in Type 2 Diabetic Mice

**Figure 7.**
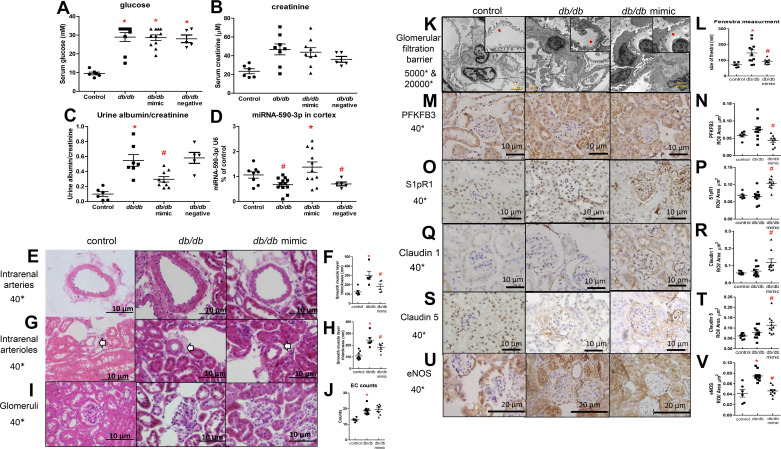
Serum glucose level (*A*), serum creatinine level (*B*), urine albumin/creatinine ratio (*C*), and miRNA-590-3p expression in renal cortex of the left kidney (*D*) in db/db mice administered with miRNA-mimics and its negative control (negative). Control mice, *n* = 6 (male:female 3:3); diabetic control mice, *n* = 9 (male:female 5:4); diabetic mice administered with mimics, *n* = 11 (male:female 7:4); diabetic mice administered with negative control *n* = 4 (male:female 2:2); *P < 0.05 compared with control mice. #*P* < 0.05 compared with db/db control using one-way ANOVA. hematoxylin-eosin (H&E) staining (*E*) and measurement of smooth muscle thickness of intrarenal arteries (*F*), H&E staining (*G*), and measurement of smooth muscle thickness of intrarenal arterioles (indicated with white arrow) (*H*), H&E staining of glomeruli (*I*), and counts of glomerular endothelial cells of the left kidney (*J*) in control and db/db mice treated without and with miRNA-590-3p mimics. *K*: glomerular filtration barrier in control and db/db mice treated without and with miRNA-590-3p mimics, and (*insets*) high magnification (20,000*) of glomerular endothelial fenestra indicated with red arrows. *L*: measurement of fenestra. *P < 0.05 compared with control mice. Protein (*M*, *O*, *Q*, *S*, and *U*) presence and (*N*, *P*, *R*, *T*, and *V*) quantification of phosphofructokinase/fructose bisphosphatase 3 (PFKFB3, *M* and *N*), receptor for sphingosine 1-phosphate (S1pR1, *O* and *P*), Claudin 1 (*Q* and *R*), Claudin 5 (*S* and *T*), and nitric oxide synthase, endothelial (eNOS, *U* and *V*) in glomeruli of the left kidney in control (*left*), db/db mice (*middle*), and db/db mice treated with miRNA-590-3p mimics (*right*). *P < 0.05 compared with control mice. #*P* < 0.05 compared with diabetic mice using one-way ANOVA.

Compared with control mice, 10-wk-old *db/db* mice had significantly higher serum glucose levels, but comparable serum creatinine levels (Fig. 7, *A* and *B*). Urinary albumin/creatinine ratios were significantly higher in *db/db* mice than in control mice (Fig. 7*C*).

The expression of miRNA-590-3p in the left kidney of 10-wk-old diabetic mice was slightly reduced compared with their background controls (Fig. 7*D*). Administration of miRNA-590-3p in perirenal adipose tissue significantly increased the miRNA-590-3p levels in the renal cortex of the left kidneys (Fig. 7*D*) compared with those injected with negative control as well as those in the right kidneys (Supplemental Fig. S2*D*). Overexpressing miRNA-590-3p via perirenal adipose tissue injection did not affect their glucose levels or serum creatinine levels in type 2 diabetic mice (Fig. 7, *A* and *B*).

Hypertrophic intrarenal arteries and arterioles (Fig. 7, *E–H*), enlarged glomeruli (Fig. 7*I*), and increased counts of glomerular endothelial cells (Fig. 7*J*) were observed in diabetic mice. Electron microscopy showed both foot cell fusion and disarrangement of endothelial fenestration in diabetic glomeruli (Fig. 7, *K* and *L*). Overexpressing miRNA-590-3p restored morphological changes in intrarenal arteries, but did not markedly alter pathological changes in glomeruli (Fig. 7, *E–I*). The endothelial fenestration disarrangement, but not foot cell fusion, was improved in treated mice (Fig. 7, *K* and *L*). The protein presence of PFKFB3 was not changed in the comparison of diabetic and control mice. Compared with diabetes, miRNA-590-3p mimics treatment reduced the PFKFB3 presence in glomeruli (Fig. 7, *M* and *N*). The protein presence of S1pR1was distributed in cell nuclei in the kidneys of diabetic mice, whereas the upregulated S1pR1 protein was mainly presented in cell membranes in diabetic glomeruli treated with miRNA mimics (Fig. 7, *O* and *P*). The protein presences of the junction proteins, Claudin 1 (Fig. 7, *Q* and *R*) and Claudin 5 (Fig. 7, *S* and *T*), were enhanced in the glomeruli in diabetic mice receiving miRNA-590-3p mimics (Fig. 7, *Q–T*). The protein presence of eNOS was increased in glomeruli of diabetic mice, and was reduced in those with miRNA mimic treatment (Fig. 7, *U* and *V*). The protein presence of ANPGT2 and VEGFA, but not ANPGT1, were also increased in diabetic mice; however, miRNA-590-3p treatment did not have any effect on them (Supplemental Fig. S10).

## DISCUSSION

The present study indicates that proangiogenic GECs are a critical player in the development of diabetic nephropathy, leading to impaired glomerular filter barrier and albuminuria. Glycolysis enzyme PFKFB3 regulates S1pR1 expression in proangiogenic endothelial cells and reduces proteinuria in diabetic mice.

In the present study, restoring glomerular endothelial function, but not podocyte foot fusion, shown as reduced filopodia sprouting and stretching in vitro and normalized endothelial fenestration in vivo, reduced albuminuria in diabetic mice. These data demonstrate the critical role of GECs in maintaining the glomerular filtration barrier (4–6). In addition to its obligatory role in releasing nitric oxide and regulating local vascular tone (40–42), endothelial cells in the microvasculature also take part in initiating immune responses (21) and maintaining barrier integrity (43). The heterogeneity of glomerular endothelial cells in mice of diabetic nephropathy implies that some endothelial cells are activated in the progress of the disease. Indeed, a subcluster of glomerular endothelial cells possessing angiogenic capacity is noted in patients with diabetes and animal models, both type 1 and type 2, confirming the participation of proangiogenic glomerular endothelial cells.

Both single-cell analyses and pathological examination indicate increased counts of glomerular endothelial cells with proangiogenic features in diabetic nephropathy, implying that endothelial proliferation and probably immature angiogenesis occur in the development of diabetic nephropathy. Angiogenesis is a process involving neovessel formation from existing vessels, and, except during embryonic development, takes place under pathological conditions. An initiating step in angiogenesis is the presence of sprouting endothelial cells, including tip and stalk cells, which have higher mobility and lower presences of adhesion and junction protein than quiescent ones. The phenotypic shift of endothelial cells in microcirculation probably results in vessel leakage, thus accounting for pathological changes in diabetic complications, such as diabetic nephropathy and retinopathy.

Given the report that miRNA-590-3p exerts protection on myocardial infarction mainly by inhibiting pathological angiogenesis, together with the finding of the presence of proangiogenesis glomerular endothelial cells in the present study, further experiments were conducted to examine the role of this miRNA on proangiogenic endothelial cells in diabetic nephropathy. The findings that the expressions of miRNA-590-3p were reduced in diabetic kidneys (44) and high-glucose-stimulated glomerular endothelial cells support the involvement of miRNA-590-3p in glomerular endothelial dysfunction in diabetic nephropathy. More importantly, mimics of miRNA-590-3p in the kidney restored renal artery structure, improved glomerular endothelial function, and reduced albuminuria in diabetic mice, thereby providing evidence for the protective effects of miRNA-590-3p in microvasculature endothelial cells challenged with high glucose.

Endothelial cells favor glycolysis in energy-producing processes (8). In the present study, the PFKFB3 upregulation and increased glycated rates were observed in proangiogenic GECs challenged with high glucose, but not with mannitol. It indicates that the protein changes in the present study are attributed to high-glucose-induced detrimental effects, but not to the osmotic changes in the culture medium. It further supports the claim that proangiogenic GECs are energetic. Downregulating PFKFB3 by miRNA-590-3p mimics reduced glomerular endothelial migration and filopodia extension in the proangiogenic GECs and in diabetic glomeruli in vivo, implying the importance of the glycolytic enzyme PFKFB3 in angiogenesis. The finding in luciferase assay that miRNA-590-3p regulated PFKFB3 expression confirms the role of miRNA-590-3p in angiogenesis. Taken together, these data reinforce the critical role of the glycolytic enzyme PFKFB3 in GECs in the process of angiogenesis.

Endothelium-derived nitric oxide is the critical player in regulating local vascular tone (42). Nevertheless, diabetes-induced nitric oxide-mediated vasodilatation was various in both macro- and microvasculature (40, 41). Protein expression of eNOS, as well as its activity, is increased (45) and reduced (46) in diabetic kidneys. Deletion of eNOS diminishes estrogen-derived protective effects in female mice of diabetic nephropathy (47) and accelerates the progress in streptozotocin-induced type 1 diabetic mice (5). In the present study, the presence of eNOS was increased in diabetic mice and reduced in diabetic mice treated with miRNA mimics. Likewise, the reduced expression of eNOS, both total and activated form, was observed in proangiogenic GECs incubated with high glucose and miRNA mimics. Of note, the reduced eNOS expression and its product nitric oxide further led to reduced cyclic guanosine monophosphate (cGMP) (the second messenger of nitric oxide) and upregulated PDE3A [also known as cGMP-inhibited phosphodiesterase, mainly hydrolyzes cyclic adenosine monophosphate (cAMP)]. In turn, the increased cAMP hydrolysis downregulates PKA-mediated glycogenolysis. Thus, it implies that overexpressing miRNA-590-3p reduces immediate glucose supply and further blunts the glycolysis process in proangiogenic GECs. Neither the nitric oxide donor nor eNOS inhibitor affected the PFKFB3 and S1pR1 protein expressions; thus, the NO-cAMP-PKA-mediated glycogenolysis plays a minor role. Of note, high glucose increased PFKFB3, but not eNOS expression in proangiogenic GECs, although overexpressing miRNA-590-3p downregulated both protein expressions. The unparallel changes of eNOS and PFKFB3 proteins in proangiogenic GECs do not support the claim that eNOS increases glycolysis through upregulating PFKFB3 (48).

Angiogenesis regulators include angiopoietins (31, 32), VEGF-VEGFRs (33, 34), ataxia-telangiectasia mutated (49, 50), thrombospondin 1 (51), and S1pR1 (35–37). Indeed, in diabetic mice the presence of ANGPT2 and VEGFA were increased in kidneys of diabetic mice. Nevertheless, high-glucose stimulation did not alter angiopoietins and VEGFA expressions in cultured GECs, suggesting that angiogenesis in diabetic glomeruli is induced in a paracrine manner. Protein expressions of VEGFRs, including VEGFR1, VEGFR2, and VEGFR3, are comparable in the cultured cells, implying that canonical mechanisms underlying angiogenesis are not involved in GECs stimulated with high glucose.

Enhanced S1pR1 expression in endothelial cells is reported to protect endothelial cell barriers by preventing small molecule leakage (13, 14, 52). In the present study, inhibition of glycolysis by pharmacological and genetic approaches enhanced S1pR1 expression in proangiogenic GECs, implying that glycolysis in GECs regulates S1pR1 expression. The translocation of S1pR1 to the glomerular endothelial cell membrane, together with its enhanced expression in treated diabetic mice, demonstrates its protective effects on endothelial barriers (13, 14, 52–57). Therefore, miRNA-590-3p-regulated glycolysis increases S1pR1 expression, protects the endothelial barrier function, and reduces albuminuria in diabetic mice.

S1pR1 agonist SEW2871 and modulator FTY720 increased S1pR1 expression in proangiogenic GECs (13, 14, 52), but did not affect PFKFB3 protein expression, suggesting that S1pR1 protein does not take part in PFKFB3 regulation.

In the present study, miRNA-590-3p mimics increased junction protein expressions, including Claudin 1 and 5, and reduced apoptosis in quiescent endothelial cells. Noted, neither junction proteins, apoptotic proteins, eNOS, nor well-studied eNOS regulators, including Akt/PKB and AMPK signaling pathways, are predicted as molecular targets of miRNA-590-3p. These data suggest that miRNA-590-3p exerts its protective effects in addition to PFKFB3 regulation. A previous report demonstrated that transfection of human renal proximal tubular cell HK-2 with miRNA-590-3p reduced the mRNA expression of CX3CL1, and this effect likely accounts for the protective effects of miRNA-590-3p against high-glucose-induced inflammatory response and injury of renal tubular cells (44). In the present study, a reduced protein presence of CX3CLI was also observed in tubular cells of *db/db* mice administered with miRNA-590-3p mimics; however, the upregulation of CX3CL1 in the glomeruli of diabetic mice was not affected by miRNA-590-3p-treatment (Supplemental Fig. S11). Thus, mechanisms underlying the miRNA-590-3p-exerted protection deserve further investigation.

Both increased proliferation and apoptosis have been reported in diabetic GECs. In the present study, high glucose stimulated cell apoptosis in endothelial cells under control but not the proangiogenic condition. High glucose alone did not affect cell viability in MTT examination in control or proangiogenic GECs. Cell viability tests, including MTT and CCK8, examine cell toxicity and proliferation, which are sometimes affected by cell metabolic activity. Thus, the inconsistent results and conclusions regarding high-glucose-elicited endothelial cell viability in the present study and literature are probably due to different stimuli, observation duration, or readouts (31, 58). In line with the aforementioned discussion, glomerular endothelial viability, migration, and angiogenesis are affected by other cells in glomeruli, probably podocytes (31, 32, 59).

**Figure 8.**
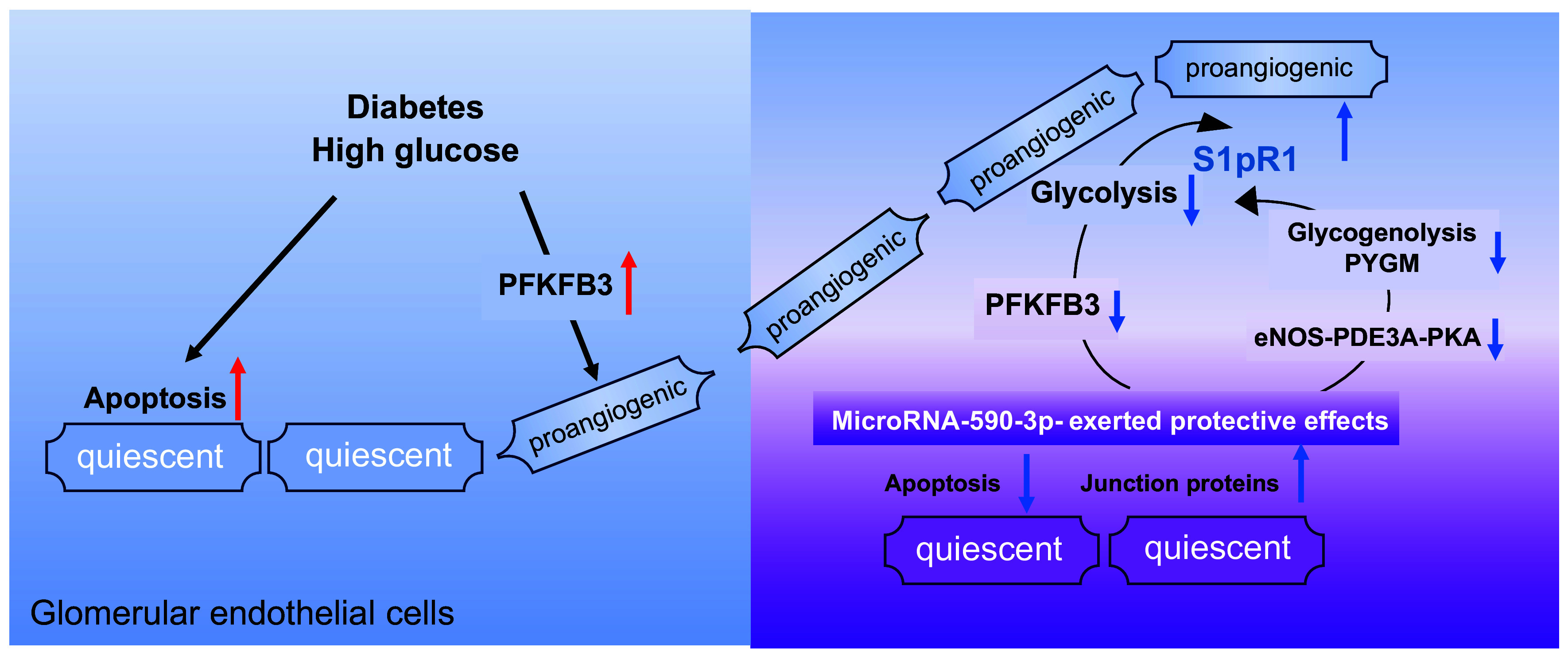
Schematic diagram illustrating miRNA-590-3p-elicited protective effects. In diabetic glomeruli, high glucose induces apoptosis in quiescent endothelial cells and increases phosphofructokinase/fructose bisphosphatase 3 (PFKFB3) expression in proangiogenic endothelial cells. Local administration of miRNA-590-3p mimics ameliorates diabetes-induced albuminuria in type 2 diabetic mice by upregulating junction proteins in quiescent endothelial cells and increasing protein presence of angiogenic modulator receptor for sphingosine 1-phosphate (S1pR1) in proangiogenic glomerular endothelial cells (GECs).

Taken together, enhanced glycolysis is crucial for proangiogenic GECs in developing diabetic nephropathy. Local administration of miRNA-590-3p increases the protein presence of angiogenic modulator S1pR1 by downregulating glycolysis in proangiogenic GECs, and increases junction protein expression in control GECs (Fig. 8). Nevertheless, detailed mechanisms underlying diabetes-induced angiogenesis in glomeruli are required for further investigation. Of importance, the multiple protective effects of miRNA-590-3p in the present study shed light on therapeutic strategy in patients of diabetic nephropathy.

## DATA AVAILABILITY

The single-cell sequencing data (GSE206015) and RNA-sequencing data (GSE219268) of glomerular cells in type 2 diabetic mice are not publicly available, but are available from the corresponding author on reasonable request.

## SUPPLEMENTAL DATA

10.6084/m9.figshare.24012852 Supplemental Figs. S1–S11: https://doi.org/10.6084/m9.figshare.24012852.

## GRANTS

This work was supported by the Zheng-Yi Scholar Scientific Research Project of Fudan University under Grant S19-15 (to K.S.), the Dual First-Class Initiation Fudan University under Grant IDF152057 (to Y.S.), and the Nature Science Foundation of Shanghai under Grant No. 22ZR1411600 (to Y.S.).

## DISCLOSURES

No conflicts of interest, financial or otherwise, are declared by the authors.

## AUTHOR CONTRIBUTIONS

S.W.S.L. and Y.S. conceived and designed research; B.Y., K.S., T.L., J.L., M.M., W.L., and Q.T. performed experiments; K.S. and J.L. analyzed data; Y.S. interpreted results of experiments; T.L. and Y.S. prepared figures; B.Y., K.S., and Y.S. drafted manuscript; T.Z., X.W., S.W.S.L., and Y.S. edited and revised manuscript; B.Y., K.S., T.L., J.L., M.M., W.L., Q.T., T.Z., X.W., S.W.S.L., and Y.S. approved final version of manuscript.
